# Papoid

**Published:** 1893-03

**Authors:** 


PAPOID.—There are many members of our profession who, after years of trial, have almost lost faith in the Pepsins; and especially is this true in those cases of so-called atonic dyspepsia, in which Pepsin has been relied on and found wanting, and there are thousands of such cases which daily present themselves to physicians for treatment. Frequently the statement is made: "Doctor, I have taken all sorts of Pepsin and without relief;" but the doctor says, "here is a new preparation of Pepsin which, I am sure, will benefit you." The patient takes his prescription and later on takes his Pepsin; he soon returns with the same old story; his digestion is no better, and he is feeling worse, if possible, than before. It is this result which, as I said previously, has caused a large number of the thinking members of our profession to lose faith in Pepsin, and they only continue to prescribe it in a routine sort of way, largely from habit, but more especially because they have been unable to find anything better. To such I say, try Papoid and you will not be disappointed. Herschell and Woodbury have pointed out that Papoid has greater digestive power than either Pepsin or Pancreatin, and can be used when Pepsin is contra-indicated or powerless. Experience has proven this to be true, and it may be stated without the fear of contradiction that Papoid under the conditions indicating the use of Animal Pepsin will produce some results, while animal Pepsin under the conditions indicating the use of Papoid will produce no results whatever. It may be further stated that *Papoid under Papoid conditions produces greater results than Animal Pepsin under Pepsin conditions!* Papoid is indicated in any case where there is a deficiency of the gastric juice, no matter from what cause; in gastric catarrh, acute or chronic; in cases of anaemia and general debility, productive of deficient blood supply; in chronic alcoholism, which is always accompanied by an excess of unhealthy mucus in the alimentary canal; in the vomiting of pregnancy and all irritable conditions of the stomach associated with pain and vomiting. In duodenal and intestinal indigestion, Papoid is infinitely superior to Pancreatin. These are no imaginary statements, but are based upon absolute results from practical experience, and from records that cover both negative and positive results.—*Extract from a paper by Arch Dixon, M. D., Henson, Ky.*

